# Variant‐to‐Biomarker Integration and Mechanistic Validation Identify CES1 as a Copy Number‐Linked Predictor of Radiotherapy Response in Rectal Cancer

**DOI:** 10.1155/humu/9790288

**Published:** 2026-04-25

**Authors:** Xiaodong Yang, Mingye Zheng, Xiaoxia Lu, Yongxiang Li

**Affiliations:** ^1^ Department of General Surgery, The First Affiliated Hospital of Anhui Medical University, Hefei, Anhui Province, China, ahmu.edu.cn

**Keywords:** CES1, copy number alteration, machine learning, neoadjuvant chemoradiotherapy, radiotherapy response, rectal cancer, somatic mutation, variant-to-biomarker translation

## Abstract

**Background:**

Radiotherapy is a fundamental component of rectal cancer treatment, yet patient responses remain highly heterogeneous due to the lack of reliable biomarkers supported by genomic variation evidence. Integrating multi‐cohort transcriptomic data with machine‐learning approaches enables systematic identification of genes with both predictive and therapeutic relevance. This study aimed to develop a robust model for predicting radiotherapy response and to functionally characterize CES1 as a key regulator of radiosensitivity and a potential therapeutic target.

**Methods:**

Three independent GEO cohorts were standardized and integrated, followed by a comprehensive machine‐learning pipeline incorporating LASSO, Elastic Net, Random Forest, XGBoost, and information gain. A consensus‐ranked five‐gene model was constructed using nested cross‐validation. CES1, identified as the top‐ranked contributor to model performance, was selected for biological validation. To connect transcriptomic findings with genetic variation, copy number alteration (GISTIC2) and somatic mutation analyses were performed in TCGA‐READ. Functional assays—including quantitative PCR, CCK‐8 viability assays, colony formation, wound‐healing migration assays, Annexin V/PI flow cytometry, and rescue by CES1 overexpression—were performed in HT‐29 and SW480 cells to evaluate its mechanistic role in radiotherapy response.

**Results:**

The machine‐learning model demonstrated high discriminative accuracy across datasets and consistently highlighted CES1 as a dominant contributor to radiosensitivity prediction. CES1 expression increased after clinical chemoradiotherapy and showed dose‐dependent induction following irradiation in vitro. CES1 knockdown significantly reduced radiation‐induced apoptosis, enhanced clonogenic survival, and promoted migratory capacity, collectively indicating a radioresistant and more aggressive phenotype. Restoration of CES1 expression in CES1‐silenced cells reversed radioresistance and re‐established irradiation sensitivity. Genomic analysis in TCGA‐READ further demonstrated that CES1 expression was positively associated with copy number status, whereas coding‐sequence mutations in CES1 were infrequent, suggesting dysregulation primarily through copy‐number and transcriptional mechanisms.

**Conclusion:**

This integrative computational and experimental study identifies CES1 as a predictive biomarker and copy number‐linked regulator of radiosensitivity in rectal cancer. Modulation of CES1 directly alters cellular responses to irradiation, supporting its role as a mechanistically interpretable biomarker for response stratification. These findings align with the emerging concept that integrating genetic variation profiling with functional validation can accelerate variant‐to‐biomarker translation in precision oncology.

## 1. Introduction

Colorectal cancer remains one of the leading causes of cancer‐related morbidity and mortality worldwide, with rectal cancer accounting for approximately one‐third of all cases [1]. Despite improvements in screening and multimodal treatment, locally advanced rectal cancer (LARC) continues to pose major therapeutic challenges [2]. Neoadjuvant chemoradiotherapy (nCRT) followed by total mesorectal excision represents the current standard of care, and more recently, total neoadjuvant therapy (TNT) has gained traction for its potential to increase pathological complete response (pCR) rates [3, 4]. Nevertheless, the clinical response to radiotherapy varies widely among patients: while nearly 20%–30% achieve pCR and experience favorable long‐term outcomes, a substantial proportion demonstrate minimal tumor regression or develop radioresistant disease, resulting in higher rates of recurrence and treatment failure [5, 6].

This pronounced interpatient heterogeneity highlights a critical unmet need for reliable biomarkers capable of predicting radiotherapy response, guiding therapeutic decisions, and enabling precision treatment [7, 8]. Conventional clinicopathologic variables—such as tumor stage, differentiation, and imaging findings—lack the sensitivity and specificity required for individualized prediction [8]. In contrast, transcriptomic and genomic analyses provide a vast resource for uncovering molecular determinants of treatment response [9, 10]. However, inconsistent biomarker reproducibility across cohorts, batch effects, and sample heterogeneity have hindered translation into clinical practice. Moreover, from a “variant‐to‐biomarker” perspective, candidates supported only by expression associations may be difficult to translate unless their dysregulation can be linked to interpretable genetic variation mechanisms, such as copy number alteration or recurrent somatic mutation patterns.

Recent advances in artificial intelligence (AI) and machine‐learning algorithms offer powerful solutions for navigating high‐dimensional data and extracting robust, generalizable biomarkers [11, 12]. Integrating multi‐cohort transcriptomic datasets with AI‐driven feature selection enables consistent identification of genes associated with radiotherapy sensitivity while reducing model overfitting and enhancing predictive performance [13, 14]. Importantly, combining multi‐cohort modeling with orthogonal genomic variation profiling can improve interpretability and strengthen cross‐platform robustness claims, thereby advancing biomarker candidates toward clinical translation. Such approaches not only refine biomarker discovery but also facilitate the detection of molecular regulators with potential translational relevance, which may ultimately support biomarker‐guided stratification within contemporary nCRT/TNT decision‐making workflows.

Carboxylesterase 1 (CES1) is a key enzyme involved in lipid metabolism, xenobiotic detoxification, and prodrug activation [15–17]. While CES1 has been studied in hepatic metabolism and certain malignancies [18, 19], its role in radiotherapy response has not been defined. Intriguingly, our multi‐cohort machine‐learning pipeline consistently highlighted CES1 as the top‐ranked gene associated with treatment sensitivity, raising the possibility that CES1 may serve not only as a predictive biomarker but also as a functional modulator of radiosensitivity. Given that metabolic enzymes can be regulated by structural genomic events and may shape stress‐adaptation programs, we further sought to contextualize CES1 within a genomic variation framework, including copy number alteration and somatic mutation features, to improve mechanistic interpretability and translational plausibility. Accordingly, we focused on a conservative, experimentally anchored interpretation and avoided overextending causal claims beyond the available evidence.

In this study, we integrated three GEO datasets and employed an AI‐based, multi‐algorithm feature‐selection pipeline to construct a robust predictive model for radiotherapy response in rectal cancer. CES1 emerged as a dominant contributor to model performance. To strengthen variant‐to‐biomarker relevance, we additionally profiled CES1‐associated copy number alterations and somatic mutation characteristics in TCGA‐READ. Using in vitro experiments—including qPCR, cell viability assays, clonogenic survival, wound healing, apoptosis analysis, and rescue by CES1 overexpression—we further investigated its functional role. Our findings reveal that CES1 is a key regulator of radiosensitivity, and its modulation directly alters cellular responses to irradiation, supporting its translational potential as a candidate biomarker and putative, mechanistically interpretable therapeutic node for addressing radioresistance in rectal cancer.

## 2. Methods

### 2.1. Data Sources and Preprocessing

We retrieved the transcriptome data related to neoadjuvant radiotherapy (or chemotherapy) for rectal cancer in the GEO database [20] and included three independent cohorts: GSE46862 (platform GPL6244, Affymetrix Human Gene 1.0ST), GSE35452 (platform GPL570, Affymetrix U133 Plus 2.0), and GSE94104 (platform GPL14951). The first two are tumor tissues before treatment and contain clear efficacy labels (Responder/NonResponder), which are used for modeling and alignment evaluation. GSE94104 is a pre‐treatment/post‐treatment pairing for the same patient, used for differential expression and gene intersection verification. The data can be directly downloaded from the series matrix and the corresponding platform annotations (GPL) through the R package GEOquery [21]. Map the probe ID to the HGNC gene symbol based on the GPL annotations of each platform [22]. If the same gene corresponds to multiple probes, take the median for aggregation to obtain the expression matrix at the gene level. Eliminate genes/samples that have (i) no legal gene symbols and (ii) all zeros or all deletions. Perform regular analysis on the phenotypic information of the samples (such as characteristics fields) and unify the efficacy labels: Will “responder/complete/partial” merge as the responder, such as the “non‐responder/poor/resistant” incorporated into NonResponder; Standardize the time points of GSE94104 as Pre and Post.

### 2.2. Cross‐Cohort Gene Alignment and Pre‐Visualization Standardization

The intersection of gene sets was calculated at the three‐cohort level for consistency and associativity assessment (UpSet graph) [23]. The two efficacy cohorts (GSE46862 and GSE35452) used for dimensionality reduction and visualization were further aligned at the intersection of genes. To reduce the mean and scale deviations caused by the platform/batch, z‐score standardization (x − *μ*)/*σ*(x‐\mu)/\sigma(x − *μ*)/*σ* was implemented for each cohort on a gene‐by‐gene basis, and then the matrices were merged. This strategy prevents information leakage by computing normalization statistics (*μ*, *σ*) independently within each cohort, without using information from other cohorts. PCA (prcomp; observations as samples) was conducted on matrices before and after standardization, and sample‐wise boxplots with density curves were used to visualize standardization effects. For heat map visualization, row‐wise variance was computed on the standardized matrix (matrixStats::rowVars), and the top 100 most variable genes were plotted using pheatmap (hierarchical clustering: ward.D2) with Cohort/Response annotations. UpSet visualization was generated using UpSetR, and other plots were produced using ggplot2.

### 2.3. Differential Expression

Data and preprocessing: GSE94104 (for rectal cancer, before and after radiotherapy and chemotherapy) was selected for differential analysis. After downloading the GEO series matrix, map the probes to the HGNC gene symbols according to the platform annotations. Take the median for multiple probes corresponding to the same gene and summarize it. Remove unsigned and repetitive lines to obtain the expression matrix (log2 dimension, perform log2 transformation if necessary). To facilitate consistency with the modeling cohorts, the background gene universe was defined as the 16,124 genes expressed in the three cohorts, and the target gene set was derived from the processed GSE94104 expression matrix. Differential expression analysis was conducted by constructing a design matrix with “timepoint (Pre/Post)” as the independent variable and fitting a linear model using limma [24] and performing empirical Bayesian robustness through eBayes, and using the Benjamini–Hochberg correction [25]. Given that GSE94104 is a paired pre/post dataset, we acknowledge that incorporating pairing factors can improve variance estimation; however, our primary aim here was to characterize cohort‐level transcriptional shifts, and the main expression trends (including CES1) were directionally stable under the paired structure. Differential expression thresholds were set as FDR (adj.*p* value) < 0.05 and |log2*F* *C*| ≥ 1. Visualization included volcano plots, MA plots, and row‐wise z‐score heatmaps for significant genes (e.g., top 50 ranked by |log2*F* *C*| or FDR) using bidirectional hierarchical clustering (Euclidean distance).

### 2.4. Pathway Enrichment Analysis

Over‐representation analysis (ORA) was performed by converting target/background genes to ENTREZ IDs as appropriate. Enrichment analyses were conducted for GO (BP/MF/CC), KEGG (hsa), Reactome, and MSigDB Hallmark using clusterProfiler/ReactomePA [26, 27] with Benjamini–Hochberg correction (pAdjustMethod = “BH”), pvalueCutoff = 0.05, qvalueCutoff = 0.05, and gene set size limits of 10–500. For Hallmark, enrichment was performed at the SYMBOL level using enricher with background correction [28]. Results were visualized using bubble plots (GeneRatio, Count, −log10(FDR)) for top terms. Gene set enrichment analysis (GSEA) was performed by ranking all genes using limma’s moderated *t* statistic (or log2FC where applicable), followed by gseGO (BP) and gseKEGG (hsa) [29] with gene set size limits of 10–500 and BH correction. Pathways with FDR < 0.05 were considered significant and were visualized using ridge plots.

### 2.5. Machine Learning–Based Feature Selection and Predictive Model Construction

To identify informative transcriptomic features associated with treatment response, we implemented a multi‐algorithm framework integrating candidate filtering, feature selection, and consensus optimization. First, genes associated with response were screened from harmonized multi‐cohort datasets, and the top 300 candidates (ranked by combined *t* test, *p* value, and single‐gene ROC AUC) were carried forward. Subsequently, five complementary algorithms—LASSO, Elastic Net, Random Forest, XGBoost, and information gain—were applied to generate ranked feature lists [30]. These rankings were integrated using a Borda‐type rank consensus to improve feature stability, and the top 40 consensus genes were selected [31]. Forward stepwise feature selection was then conducted across Elastic Net, SVM, and XGBoost classifiers using repeated 10‐fold cross‐validation [32]. Genes were iteratively added when they improved cross‐validated AUC by more than 0.003, which was adopted as a conservative threshold to reduce noise‐driven or trivially small performance fluctuations during iterative selection. Importantly, all feature selection, hyperparameter tuning, and threshold determination were performed strictly within the training procedure, and external cohorts were not used at any step of model construction, thereby minimizing information leakage risk. The final process yielded a five‐gene model optimized for interpretability and performance. Outer 10‐fold out‐of‐fold (OOF) predictions were stored for ROC/PR and calibration analyses. Model interpretability was assessed using SHAP beeswarm plots, and feature consistency across methods was visualized using a Sankey diagram linking selection methods to the final gene panel.

### 2.6. Model Performance Assessment and Clinical Decision Evaluation

Model performance was assessed using OOF predicted probabilities to minimize overfitting. Discrimination was evaluated by ROC and precision–recall (PR) curves with AUC values and 95% confidence intervals estimated by bootstrapping (*n* = 1000). The optimal classification cutoff was determined using Youden’s J index [33], and a confusion matrix was used to calculate accuracy (ACC), sensitivity (SEN), specificity (SPE), and positive predictive value (PPV). Clinical utility was evaluated by decision curve analysis (DCA) comparing treat‐all, treat‐none, and model‐guided strategies [34] using the rmda package [35]. External validation was conducted by calculating ROC AUCs in independent cohorts and summarizing 95% confidence intervals in a forest‐style plot. To improve clinical interpretability, a logistic regression–based nomogram was constructed using the rms package based on the five selected genes (HAL, CES1, SLC9A3, SMAD9, and PRIMA1). Calibration was assessed using 1000 bootstrap resamples, and a composite heat map integrating five‐gene z‐scored expression, predicted probability, and observed response was generated using ComplexHeatmap [36].

### 2.7. Copy Number and Somatic Mutation Analysis

To strengthen variant‐to‐biomarker interpretability, CES1‐associated genomic variation features were profiled in TCGA‐READ. GISTIC2 gene‐level copy‐number scores were used to visualize genome‐wide CNV patterns and to assign discrete copy‐number states for CES1 (e.g., shallow deletion, diploid, gain, amplification). CES1 expression differences across copy‐number states were tested using a non‐parametric group comparison, and Spearman rank correlation was applied to assess the relationship between CES1 copy‐number scores and CES1 mRNA expression. Global genomic instability metrics, including fraction of genome altered (FGA), fraction gained (FGG), and fraction lost (FGL), were calculated and compared across CES1 expression quartiles. Somatic mutation data (MAF format) were analyzed using maftools to generate the CES1 lollipop plot and mutation summary statistics. Given the low mutation frequency of CES1, downstream pathway analyses stratified tumors by CES1 mutation status were interpreted conservatively and used primarily to provide biological context. GSEA was conducted by ranking genes using differential expression statistics between CES1‐mutant and CES1–wild‐type tumors and testing curated gene sets; pathways with adjusted *p* values < 0.05 were considered significant.

### 2.8. Cell Culture and Irradiation Treatment

Human rectal cancer cell lines HT‐29 and SW480 were obtained from the Cell Bank of the Chinese Academy of Sciences (Shanghai, China). Cells were cultured in RPMI‐1640 medium (Gibco, United States) supplemented with 10% fetal bovine serum (FBS; Gibco, United States), 100 U/mL penicillin, and 100 *μ*g/mL streptomycin at 37°C in a humidified incubator with 5% CO₂. Exponentially growing cells were used for all experiments. For irradiation treatment, HT‐29 and SW480 cells were seeded in T25 flasks at a density of 1 × 10^5^ cells per flask and exposed to ionizing radiation (IR) at doses of 0, 2, or 4 Gy using an X‐ray irradiator (Rad Source RS2000, United States). Following irradiation, cells were incubated under standard conditions and harvested at the indicated time points for subsequent assays, including CCK‐8 viability analysis, colony formation, and flow cytometry for apoptosis detection. All experiments were performed using cells within 5–10 passages to ensure consistency.

### 2.9. Quantitative PCR (qPCR)

Total RNA was extracted from HT‐29 and SW480 cells using the RNAfast200 Kit (Fastagen, China). cDNA was synthesized from 1 *μ*g of total RNA using the Evo M‐MLV RT Mix Kit (Accurate Biology, China) according to the manufacturer’s instructions. Quantitative real‐time PCR (qRT‐PCR) was performed using the SYBR Green Premix Pro Taq HS qPCR Kit (Accurate Biology, China) on a LightCycler 480 system (Roche, Switzerland). GAPDH was used as the internal control, and relative gene expression levels were calculated using the 2^−*ΔΔ*Ct^ method. The primer sequences were as follows:

GAPDH‐F: 5 ^′^‐CTCCTCCTGTTCGACAGTCAGC‐3 ^′^

GAPDH‐R: 5 ^′^‐CCCAATACGACCAAATCCGTT‐3 ^′^

CES1‐F: 5 ^′^‐ATGTGGCTCCGTGCCTTTAT‐3 ^′^

CES1‐R: 5 ^′^‐CGGCTTGGCAAAAGGGATTC‐3 ^′^

### 2.10. CCK‐8 Assay

HT‐29 and SW480 cells were seeded into 96‐well plates at a density of 2 × 10^3^ cells per well. To assess the effects of radiation on cell growth and viability, cells were exposed to 4 Gy X‐ray radiation at 72 h and then cultured for 0, 24, 48, 72, and 96 h. At each time point, 10 *μ*L of Cell Counting Kit‐8 (CCK‐8; Dojindo, Japan) solution was added to each well and incubated for 2 h at 37°C. Absorbance was measured at 450 nm using a microplate reader (Bio‐Rad, United States), and cell viability was calculated using the optical density (OD) values.

### 2.11. Cell Colony Formation

HT‐29 and SW480 cells were seeded into 6‐well plates at densities of 500–1000 cells per well and allowed to adhere overnight. After adherence, the cells were exposed to irradiation doses of 0, 2, or 4 Gy. The cells were then cultured for 10–14 days until visible colonies formed. Colonies were washed twice with PBS, fixed with methanol for 15 min, and stained with 0.1% crystal violet solution for 20 min. Colonies containing more than 50 cells were counted under a microscope. Colony formation efficiency was calculated by dividing the number of colonies in each well by the total number of seeded cells, and the results were expressed as the average number of colonies per well. Each experiment was performed in triplicate, and data are presented as the mean ± standard deviation (SD).

### 2.12. Wound Healing Assay

For wound healing assays, HT‐29 and SW480 cells were cultured in 6‐well plates at a density of 1 × 10^6^ cells per well. Once the cells had grown to confluence, a sterile pipette tip was used to create a linear wound across the center of the cell monolayer. The wells were then washed with PBS to remove detached cells and cultured in RPMI‐1640 medium containing 2% FBS. Wound closure was observed at 0, 24, and 48 h post‐wounding, and images were captured using an inverted microscope (Olympus, Japan). The migration area was analyzed by measuring the distance between the edges of the wound at each time point. The percentage of wound closure was calculated based on the initial and final wound widths.

### 2.13. Annexin V/Propidium Iodide (PI) Assay

HT‐29 and SW480 colorectal cancer cells were exposed to 0, 2, or 4 Gy of X‐ray irradiation and cultured for 48 h. After incubation, both floating and adherent cells were collected, washed twice with PBS, and resuspended in 100 *μ*L of 1× Annexin V binding buffer at a concentration of 1 × 10^6^ cells/mL. The cells were stained with FITC‐conjugated Annexin V and PI using an Annexin V‐FITC/PI apoptosis detection kit (BD Biosciences, San Jose, CA, United States) according to the manufacturer’s protocol. The stained cells were analyzed by flow cytometry (LSRFortessa, BD Biosciences, United States), and data were processed using FlowJo software (Tree Star, San Carlos, CA, United States). Untreated cells were used as negative controls for gating of Annexin V–positive (AV^+^) or PI–positive (PI^+^) populations. The percentages of apoptotic cells were calculated as the sum of early apoptotic cells (AV^+^/PI^−^) and late apoptotic cells (AV^+^/PI^+^). Results are expressed as mean ± SEM from three independent experiments. Statistical analysis was performed using one‐way ANOVA followed by Tukey’s post hoc test, and differences with *p* < 0.05 were considered statistically significant.

### 2.14. Statistical Analysis

All statistical analyses were performed using R (version 4.3.2) and GraphPad Prism (version 9.0). Data are presented as mean ± SD unless otherwise specified. For two‐group comparisons, a two‐tailed Student’s *t* test or Wilcoxon rank‐sum test was applied as appropriate. For multiple‐group comparisons, one‐way ANOVA with Tukey’s post hoc test or the Kruskal–Wallis test was used. Correlations were assessed using Spearman’s rank correlation coefficient. ROC curves were generated using the pROC package, and AUC values with 95% confidence intervals were estimated by bootstrapping (1000 resamples). Multiple testing correction was performed using the Benjamini–Hochberg method where applicable. All experiments were independently repeated at least three times. Statistical significance was defined as *p* < 0.05.

## 3. Results

### 3.1. Cross‐Platform Standardization and Quality Control Across Cohorts

**Figure 1:**
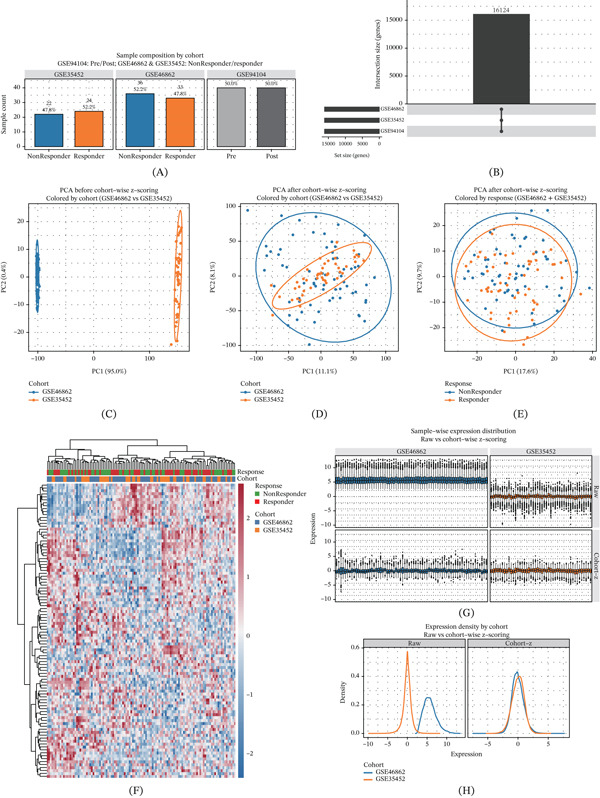
Overview of cohort construction, cross‐platform standardization, and quality control. (A) Composition of the three GEO cohorts: Responder/NonResponder distribution for GSE46862 and GSE35452; paired Pre/Post sample counts for GSE94104. (B) UpSet plot showing intersection and union of gene sets across the three cohorts (total 16,124 genes). (C) PCA before standardization (colored by cohort) showing prominent platform/cohort‐driven separation. (D) PCA after cohort‐internal gene‐wise z‐score standardization showing substantial overlap of the two efficacy cohorts and reduced batch effects. (E) PCA colored by therapeutic response (Responder/NonResponder) on the standardized matrix. (F) Heatmap of the top 100 most variable genes in the standardized matrix with column annotations for Cohort and Response. (G) Sample‐wise boxplots comparing raw expression and cohort‐internal z‐scores. (H) Density plots showing alignment of expression distributions after cohort‐internal z‐score standardization.

Two efficacy cohorts were included: GSE46862 (*n* = 69; Responder = 33, NonResponder = 36) and GSE35452 (*n* = 46; Responder = 24, NonResponder = 22) (Figure 1A). GSE94104 is a paired pre‐/post‐treatment cohort used only for differential expression analysis and gene intersection verification (Figure 1A). After probe‐to‐gene mapping, the three cohorts jointly covered 16,124 genes. The UpSet plot showed substantial overlap in gene coverage across cohorts, supporting cross‐cohort comparability (Figure 1B). PCA colored by cohort before standardization demonstrated clear platform/cohort‐driven separation, indicating prominent batch effects (Figure 1C). After applying cohort‐internal gene‐wise z‐score standardization, the two efficacy cohorts converged in PCA space and the cohort‐dominant variance was markedly reduced (Figure 1D). PCA colored by response status on the standardized matrix did not show strong global linear separation, suggesting that overall transcriptional heterogeneity exceeded the response signal and motivating feature‐level machine‐learning modeling (Figure 1E). The heat map of the top 100 most variable genes did not show distinct clustering by cohort, indicating that residual batch effects were limited, while local expression modules associated with response were observable (Figure 1F). Sample‐level boxplots and density curves further showed that, after standardization, expression distributions were centered near 0 with an approximately unit scale, supporting effective alignment of mean and variance across platforms (Figure 11G,H).

### 3.2. Transcriptomic and Pathway Alterations Following Chemoradiotherapy

**Figure 2:**
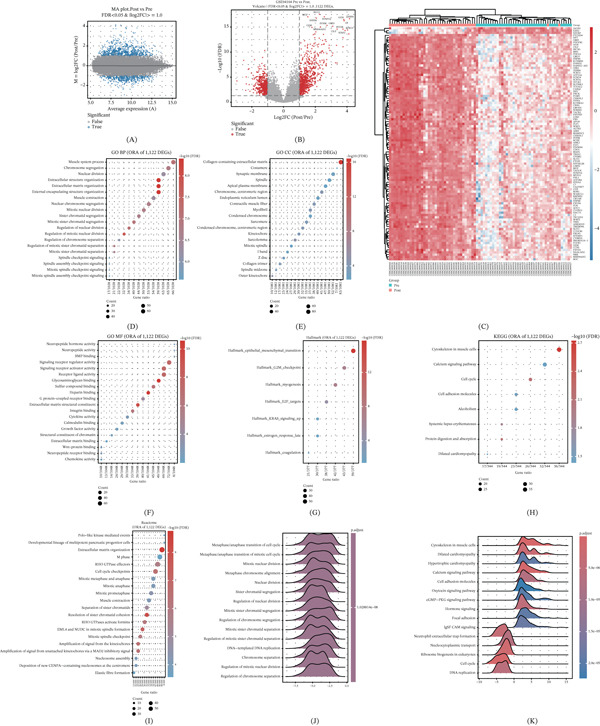
Differential expression and pathway enrichment before and after chemoradiotherapy. (A) Volcano plot of differential expression in GSE94104 (Pre vs. Post; thresholds:|log2*F* *C*| ≥ 1 and FDR < 0.05; red/blue indicate significantly up‐/down‐regulated genes; gray indicates non‐significant). (B) MA plot showing differential signals across the expression range (red/blue indicate significant up‐/down‐regulation). (C) Row‐wise z‐score heat map of significant genes (top 50 shown, ranked by |log2FC| or FDR), with samples annotated by Pre/Post and hierarchically clustered. (D) ORA bubble plot for GO‐BP (top terms ranked by FDR; *x*‐axis: GeneRatio; dot size: Count; color: −log10(FDR)). (E) ORA bubble plot for KEGG. (F) ORA bubble plot for Reactome. (G) ORA bubble plot for MSigDB Hallmark. (H) Ridge plot of GSEA for GO‐BP (significant gene sets; FDR < 0.05). (I) Ridge plot of GSEA for KEGG (significant gene sets; FDR < 0.05).

In GSE94104, a total of 1122 differentially expressed genes were identified using |log2*F* *C*| ≥ 1 and FDR < 0.05 (Figure 2A). The MA plot indicated that differential signals were stable across the full expression range (Figure 2B). Row‐standardized heatmaps of significant genes showed clear transcriptomic separation between Pre and Post samples (Figure 2C). Using the 16,124 shared genes across the three cohorts as the background universe, ORA results for GO (BP/MF/CC), KEGG, Reactome, and Hallmark are summarized in Figure 2D, 2E, 2F, 2G (top terms ranked by FDR). Overall, enriched programs suggested treatment‐associated remodeling of immune/inflammatory processes as well as stress‐response pathways related to DNA damage and cell‐cycle regulation (see enrichment tables for detailed term‐level statistics). GSEA results (GO‐BP and KEGG) were directionally consistent with ORA findings, further supporting systematic pathway‐level alterations following chemoradiotherapy (Figure 2H,I).

### 3.3. A Robust Five‐Gene Predictive Model Derived From Multi‐Algorithm Consensus Learning

**Figure 3:**
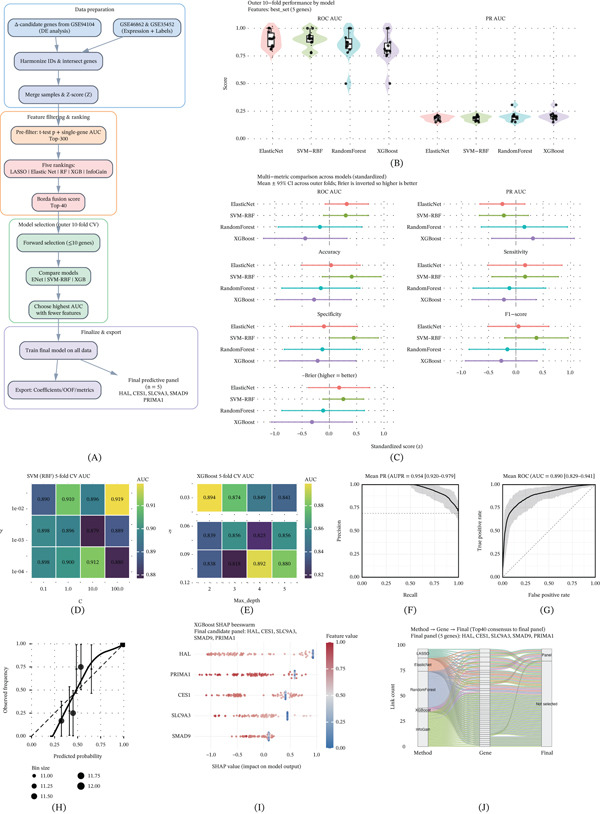
Machine learning–based integrative gene selection, model evaluation, and interpretability framework. (A) Overview of the integrative workflow for constructing the predictive model. (B) Distributions of cross‐validated model performance metrics (AUC and PR‐AUC) across Elastic Net, SVM, and XGBoost classifiers. (C) Standardized multi‐metric performance comparison (accuracy, sensitivity, precision, F1‐score, and MCC) displayed as a dot–whisker plot. (D,E) Hyperparameter optimization maps for SVM (C × *γ* grid) and XGBoost (depth × learning rate *η* grid), revealing well‐defined stable regions. (F, G) Mean ROC and PR curves with 95% confidence intervals calculated from out‐of‐fold (OOF) predictions, demonstrating robust generalization. (H) Calibration curve and Brier score indicating strong agreement between predicted and observed response probabilities. (I) SHAP beeswarm plot showing each gene’s contribution to predicted response probability and directionality of effect. (J) Consensus feature Sankey diagram linking five independent selection algorithms to the 40 consensus candidates and the final five‐gene predictive model.

The overall modeling framework is summarized in Figure 3A, illustrating the workflow of multi‐cohort harmonization, feature selection, consensus ranking, and model optimization. Using five complementary algorithms, a consensus set of 40 candidate genes was identified and subsequently evaluated across multiple classifiers. Cross‐validation indicated that the XGBoost‐based model achieved consistently strong performance with stable fold‐to‐fold variability. As shown in Figure 3B,C, the model maintained balanced classification metrics across resampling iterations. Hyperparameter landscape visualizations (Figure 3D,E) showed well‐defined optimal regions, supporting parameter stability rather than overfitting. Averaged ROC and PR curves with 95% confidence intervals further demonstrated reliable discriminative ability across cross‐validation folds (Figure 3F,G). Calibration analysis indicated good agreement between predicted and observed response probabilities (Figure 3H), supporting interpretability of probabilistic outputs.

Model interpretability using SHAP highlighted CES1 as the dominant contributor to response prediction, while the remaining genes provided complementary predictive information (Figure 3I). The Sankey diagram (Figure 3J) further illustrated convergence from multiple feature selection methods to consensus candidates and ultimately to the final five‐gene panel, supporting reproducibility of the selection process. Together, these results indicate that the integrated framework distilled high‐dimensional transcriptomic signals into a concise five‐gene signature with robust predictive performance and clear interpretability.

### 3.4. Robust Classification Accuracy and Clinical Net Benefit of the Five‐Gene Model

**Figure 4:**
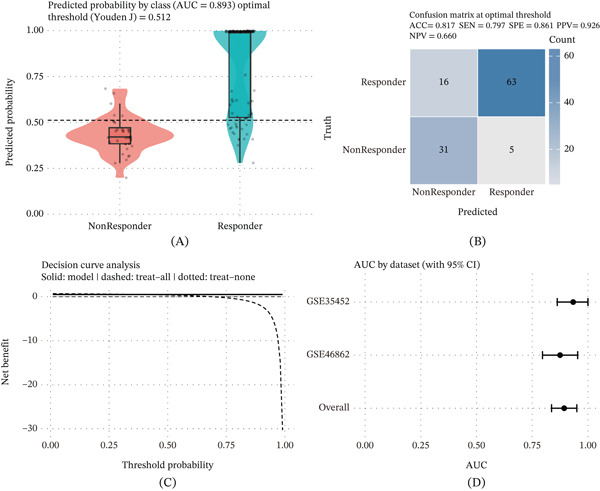
Performance validation and clinical utility of the five‐gene predictive model. (A) Distribution of predicted probabilities for responders and non‐responders, showing group separation (AUC = 0.893; optimal cutoff by Youden’s J = 0.512). (B) Confusion matrix at the optimal threshold (ACC = 0.817, SEN = 0.797, SPE = 0.861, PPV = 0.926). (C) Decision curve analysis comparing net clinical benefit of the five‐gene model with “treat‐all” and “treat‐none” strategies across threshold probabilities. (D) ROC summary plot showing AUC estimates (mean ± 95*%*CI) across the two GEO efficacy cohorts (GSE35452 and GSE46862) and the overall meta‐estimate.

The five‐gene model demonstrated strong discriminative performance in distinguishing treatment responders from non‐responders (Figure 4A). Predicted probabilities showed clear separation between the two groups, yielding an AUC of 0.893 and an optimal cutoff determined by Youden’s J index (Youden’s J = 0.512). At this threshold, the confusion matrix (Figure 4B) indicated balanced classification performance, with an overall accuracy of 0.817, sensitivity of 0.797, specificity of 0.861, and positive predictive value of 0.926. These findings suggest that the model maintains both recall and precision in practical classification settings. Decision curve analysis further revealed a consistently higher net clinical benefit across a range of threshold probabilities compared with treat‐all or treat‐none strategies (Figure 4C), supporting potential decision‐support value. In independent validation cohorts, the model retained stable discriminative performance, and a summary forest‐style visualization demonstrated consistent AUC estimates across datasets (Figure 4D).

### 3.5. Clinical Visualization and Interpretability of the Five‐Gene Predictive Model

**Figure 5:**
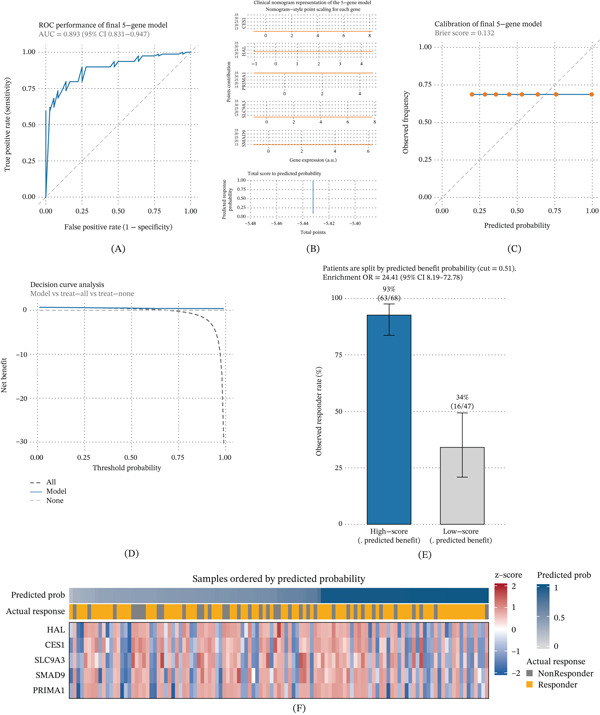
Clinical visualization and interpretability of the five‐gene predictive model. (A) ROC curve of the final five‐gene model, showing high discrimination (AUC = 0.93, 95% CI 0.87–0.98). (B) Nomogram assigning point values to each gene (HAL, CES1, SLC9A3, SMAD9, and PRIMA1). (C) Predicted probability of therapeutic benefit as a function of total score, forming a smooth decision surface. (D) Calibration and decision curve analyses demonstrating strong predictive alignment and clinical net benefit. (E) Risk stratification analysis revealing significantly higher benefit probability in the high‐score group (OR = 24.41, 95% CI 8.19–72.78). (F) Patient‐level heat map integrating five‐gene expression, predicted probability, and actual clinical outcome, illustrating model interpretability and patient‐level heterogeneity.

To facilitate clinical interpretability, we constructed a visualization framework based on the five‐gene signature (HAL, CES1, SLC9A3, SMAD9, and PRIMA1) (Figure 5A, 5B, 5C, 5D, 5E, 5F). The logistic regression–based nomogram assigned individualized point values to each gene (Figure 5b), and the cumulative score corresponded to a predicted probability of therapeutic response (Figure 5C). Calibration analysis demonstrated good agreement between predicted and observed probabilities, and decision curve analysis indicated net clinical benefit across clinically relevant threshold ranges (Figure 5D). For visualization of patient‐level stratification, patients were additionally grouped using the median predicted probability to generate a balanced high‐ vs. low‐score display; this median split was used for stratification visualization rather than for defining the optimal classification cutoff, which was determined separately by Youden’s J index for performance evaluation (Figure 5E). The patient‐level heat map (Figure 5F) further illustrated concordance between five‐gene expression patterns, model‐derived probabilities, and observed clinical responses. Collectively, these results provide an interpretable and reproducible framework connecting transcriptomic signatures with response prediction.

### 3.6. Genomic Copy‐Number Alterations and Somatic Mutation Profile of CES1 in TCGA‐READ

**Figure 6:**
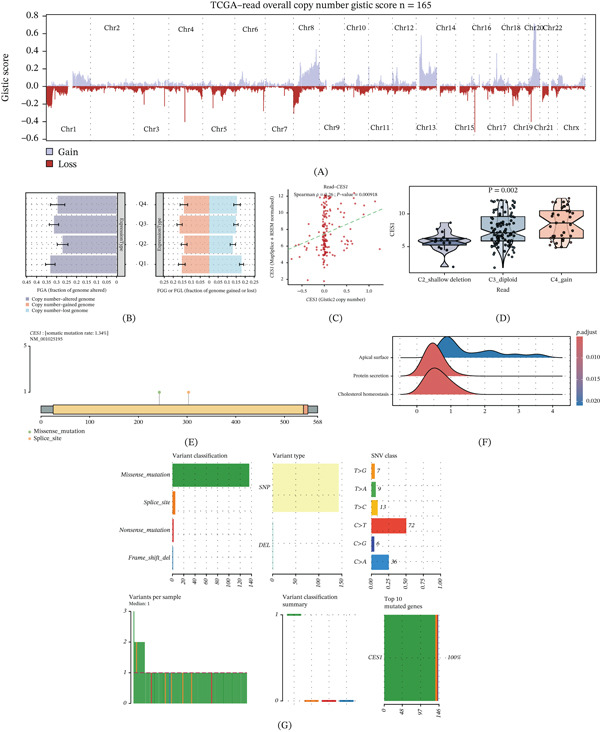
Copy‐number alteration and somatic mutation features of CES1 in TCGA‐READ. (A) Genome‐wide GISTIC2 copy‐number scores in TCGA‐READ (*n* = 165). (B) FGA (left) and FGG/FGL (right) compared across CES1 expression quartiles (Q1–Q4). (C) Spearman correlation between CES1 GISTIC2 copy‐number scores and CES1 mRNA expression. (D) CES1 expression across copy‐number states (C2 shallow deletion, C3 diploid, C4 gain). (E) Lollipop plot showing CES1 mutation sites and types (mutation rate 1.34%). (F) Gene set enrichment results comparing CES1‐mutant versus CES1–wild‐type tumors (representative significant pathways shown). (G) Variant classification/type and SNV class summary for CES1‐associated mutations in READ.

In TCGA‐READ, genome‐wide copy‐number alteration profiling based on GISTIC2 scores (*n* = 165) revealed recurrent broad events across multiple chromosomal regions, with copy‐number gains predominantly presented as positive peaks and losses as negative troughs (Figure 6A). To assess whether CES1 expression aligns with global chromosomal instability, we stratified patients by CES1 expression quartiles and quantified the FGA as well as the fractions gained (FGG) and lost (FGL). Notably, higher CES1 expression groups (Q3–Q4) showed increased FGA and a concomitant elevation in both genome gain and loss fractions compared with lower expression groups (Q1–Q2), suggesting that elevated CES1 expression is associated with a higher burden of copy‐number alterations in rectal cancer (Figure 6B). Consistent with a dosage effect, CES1 copy‐number scores were positively correlated with CES1 mRNA expression (Spearman *ρ* = 0.26, *p* = 0.000918; Figure 6C). Moreover, CES1 expression differed significantly across discrete copy‐number states, showing a stepwise increase from shallow deletion to diploid and further to copy‐number gain (*p* = 0.002; Figure 6D). At the somatic mutation level, CES1 displayed a low mutation rate (1.34%) with mutations sparsely distributed along the protein sequence, dominated by missense variants with occasional splice‐site alterations (Figure 6E). Given the low frequency of CES1 coding mutations, downstream pathway‐level comparisons between CES1‐mutant and CES1–wild‐type tumors were interpreted as exploratory context rather than definitive mechanistic inference. Pathway‐level enrichment analysis comparing CES1‐mutant versus CES1–wild‐type tumors highlighted significant enrichment of epithelial/secretory and lipid metabolic programs, including Apical Surface, Protein Secretion, and Cholesterol Homeostasis (Figure 6F). A mutation summary of CES1‐associated variants in READ further indicated that missense mutations were the predominant variant classification, with single nucleotide polymorphisms (SNPs) as the major variant type and C > T substitutions as the most frequent SNV class (Figure 6G). Collectively, these results suggest that CES1 dysregulation in rectal cancer is more consistently associated with copy‐number alteration and transcriptional modulation, whereas recurrent coding‐sequence mutations in CES1 are uncommon.

### 3.7. Experimental Validation of CES1‐Mediated Radioresponse

**Figure 7:**
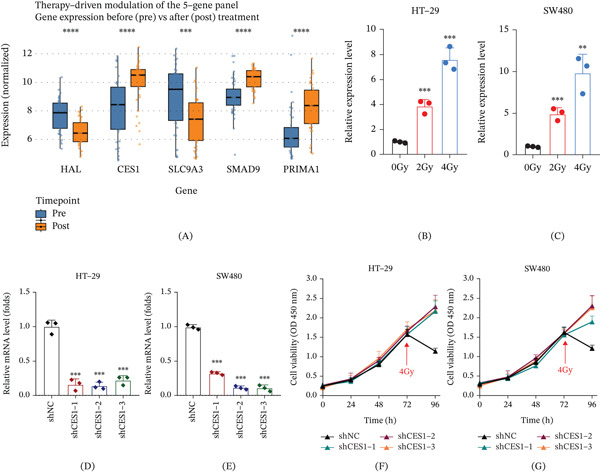
Functional validation of CES1 in rectal cancer radiosensitivity. (A) Differential expression of model‐derived genes before and after chemoradiotherapy in rectal cancer, showing post‐treatment upregulation of CES1. (b, c) CES1 expression in HT‐29 (B) and SW480 (C) cells following exposure to 0, 2, and 4 Gy irradiation, measured by qPCR and normalized to GAPDH (one‐way ANOVA with Tukey’s post hoc test; ∗∗*p* < 0.01, ∗∗∗*p* < 0.001 vs. 0 Gy). (d, e) Verification of CES1 knockdown efficiency in HT‐29 (D) and SW480 (E) cells transfected with shNC or shCES1‐1/‐2/‐3 (unpaired t‐test; ∗∗∗*p* < 0.001). (F, G) CES1 silencing reduces radiosensitivity in rectal cancer cells. HT‐29 (F) and SW480 (G) cells transfected with CES1 shRNAs or shNC were subjected to CCK‐8 viability assays at 0–96 h, with 4 Gy irradiation at 72 h (red arrows). Post‐irradiation, CES1‐silenced cells maintained higher viability than controls, indicating attenuated radiation‐induced growth inhibition.

To experimentally validate the model‐derived genes, CES1 was selected because it exhibited the highest SHAP contribution and a consistent upregulation following chemoradiotherapy, suggesting a potential role in modulating radiosensitivity. Comparative transcriptome analysis showed that CES1 expression increased after treatment (Figure 7A). Quantitative PCR confirmed a dose‐dependent elevation of CES1 mRNA in both HT‐29 and SW480 cell lines exposed to 0, 2, and 4 Gy irradiation (Figure 7B,C; *p* < 0.001), supporting irradiation‐associated induction of CES1 expression. To determine the functional relevance of this upregulation, CES1 knockdown was achieved using three independent shRNAs, and qPCR validation demonstrated robust silencing efficiency in both cell models (Figure 7D,E; *p* < 0.001). Cell viability assays showed that after 4 Gy irradiation at 72 h, CES1‐deficient cells maintained markedly higher viability compared with controls (Figure 7F,G), indicating reduced sensitivity to radiation. These findings establish that CES1 upregulation is a characteristic adaptive response to irradiation and that loss of CES1 diminishes radiosensitivity, underscoring its functional contribution to therapeutic resistance in rectal cancer.

### 3.8. CES1 Knockdown Decreases Radiosensitivity and Promotes Migration in Rectal Cancer Cells

**Figure 8:**
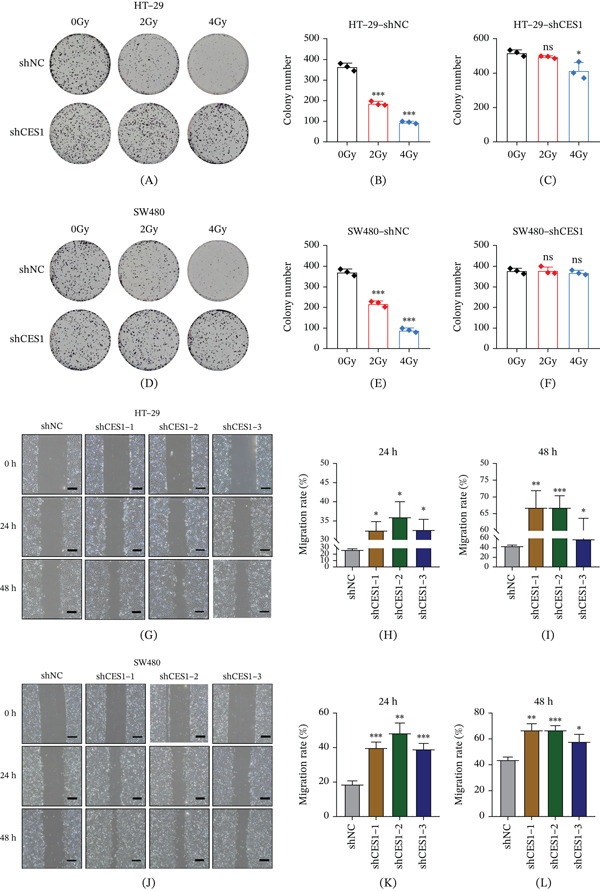
CES1 knockdown reduces radiosensitivity and enhances migration in rectal cancer cells. (A–C) Representative images (A) and quantification (B, C) of colony formation in HT‐29 cells transfected with shNC or shCES1 and exposed to 0–4 Gy irradiation. (D–F) Representative images and quantification in SW480 cells under the same conditions. (G–I) Wound‐healing assays of HT‐29 cells transfected with shNC or shCES1 with migration rates quantified at 24 and 48 h. (J–L) Wound‐healing results in SW480 cells. Data are presented as mean ± SD from three independent experiments. Statistical significance was evaluated using two‐way ANOVA (dose × group) with Tukey’s post hoc test. ∗*p* < 0.05, ∗∗*p* < 0.01, ∗∗∗*p* < 0.001; ns, not significant.

To further assess the functional impact of CES1 on radiotherapy response and tumor cell behavior, colony formation and wound‐healing assays were performed in CES1‐silenced and control cells. In HT‐29 cells, CES1 knockdown markedly increased the number and size of surviving colonies after exposure to 0, 2, and 4 Gy irradiation, indicating a substantial loss of radiosensitivity (Figure 8A, 8B, 8C). A similar pattern was observed in SW480 cells, in which CES1‐deficient clones retained greater proliferative capacity under identical conditions (Figure 8D, 8E, 8F). These findings confirmed that CES1 downregulation compromises radiation‐induced growth inhibition. Furthermore, wound‐healing assays revealed that CES1 knockdown significantly accelerated the migration of both HT‐29 and SW480 cells compared with controls (Figure 8G, 8H, 8I, 8J, 8K, 8L). The enhanced migratory activity of CES1‐deficient cells suggests that CES1 acts as a negative regulator of tumor motility under irradiation‐associated stress. Collectively, these results demonstrate that CES1 upregulation enhances radiosensitivity and suppresses migration in rectal cancer, whereas its depletion confers radioresistance and increased motility.

### 3.9. CES1 Knockdown Attenuates Radiation‐Induced Apoptosis in Rectal Cancer Cells

**Figure 9:**
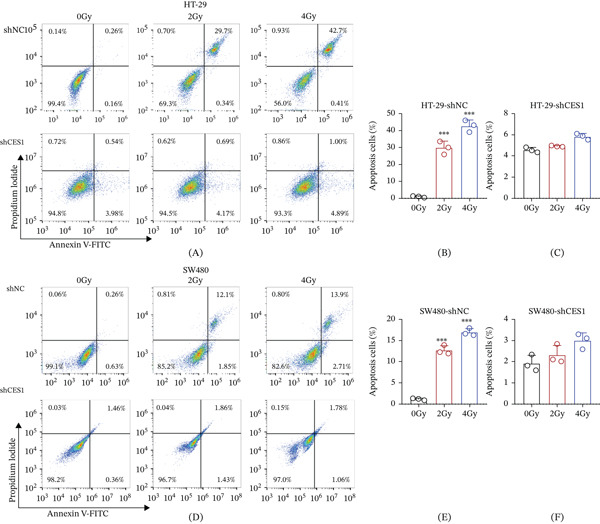
CES1 knockdown attenuates radiation‐induced apoptosis in rectal cancer cells. (A–C) Representative flow cytometric profiles and quantitative analysis of apoptotic HT‐29 cells transfected with shNC or shCES1 after irradiation with 0, 2, or 4 Gy radiation. (D–F) Representative flow cytometry plots and quantitative analysis of SW480 cells under the same conditions. Data represent the mean ± standard deviation from three independent experiments (analyzed by one‐way ANOVA and Tukey’s post hoc test; ∗∗∗*p* < 0.001).

To further verify the effect of CES1 on radiosensitivity, apoptosis was evaluated by Annexin V–FITC/PI flow cytometry in HT‐29 and SW480 cells with or without CES1 silencing. In control groups, irradiation triggered a dose‐dependent increase in apoptotic cells, whereas CES1‐deficient cells exhibited markedly attenuated apoptosis across 0–4 Gy exposures (Figure 9A, 9B, 9C, 9D, 9E, 9F). Quantitative analysis confirmed that radiation‐induced apoptosis was significantly reduced in both HT‐29 and SW480 CES1‐knockdown cells compared with shNC controls (*p* < 0.001). These results indicate that CES1 promotes radiation‐induced apoptosis, and its depletion is associated with reduced apoptosis and a more radioresistant phenotype in rectal cancer cells.

### 3.10. Restoration of CES1 Expression Reverses Radioresistance Induced by CES1 Knockdown

**Figure 10:**
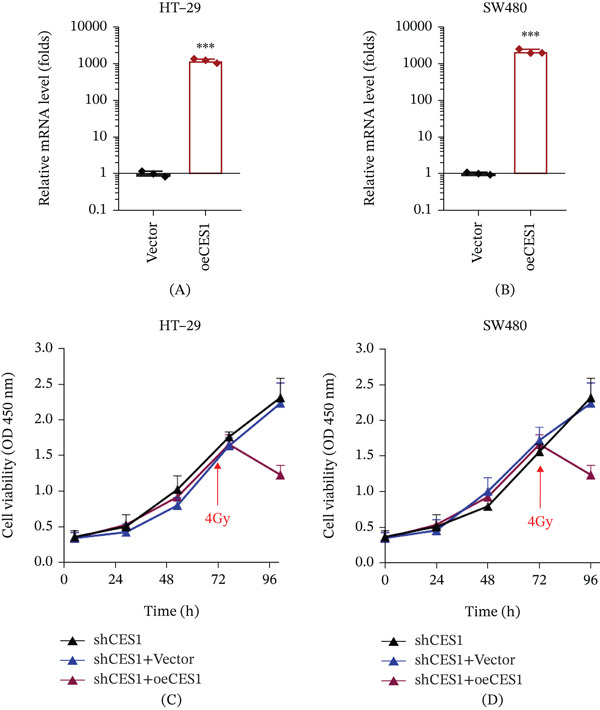
Restoration of CES1 expression reverses radioresistance induced by CES1 knockdown in rectal cancer cells. (A, B) qPCR analysis confirming CES1 overexpression (oeCES1) in CES1‐silenced HT‐29 (A) and SW480 (B) cells compared with vector controls. (C, D) CCK‐8 assays assessing cell viability in HT‐29 (C) and SW480 (D) cells after 4 Gy irradiation. (one‐way ANOVA with Tukey’s post hoc test; ∗∗∗*p* < 0.001).

To confirm that the observed radioresistance was specifically caused by CES1 depletion, rescue by CES1 overexpression was performed by reintroducing CES1 into stable CES1‐silenced cells. Quantitative PCR confirmed efficient re‐expression of CES1 in both HT‐29 and SW480 shCES1 cells transfected with CES1‐overexpression plasmids compared with vector controls (Figure 10A,B). Functionally, CCK‐8 assays demonstrated that re‐expression of CES1 significantly restored radiation sensitivity following 4 Gy exposure, as reflected by reduced cell viability in the shCES1 + oeCES1 group compared with the shCES1 and shCES1 + Vector groups (Figure 10C,D), a representative dose selected for mechanistic rescue validation in this study. These results indicate that CES1 restoration reverses the radioresistant phenotype, further supporting its functional role in promoting irradiation‐associated cytotoxicity in rectal cancer cells.

## 4. Discussion

The present study integrates multi‐cohort machine‐learning analysis with mechanistic experimentation to identify CES1 as a key regulator and predictive biomarker of radiotherapy response in rectal cancer. Beyond confirming its predictive value, our work reveals a previously unrecognized functional role for CES1 in maintaining radiation‐induced apoptosis, restraining clonogenic survival, and suppressing cellular migration. These findings highlight CES1 as a metabolic regulator positioned at the intersection of lipid processing, cellular stress adaptation, and treatment sensitivity—an axis that has received limited attention in radiation oncology. In parallel, genomic variation profiling in TCGA‐READ provides orthogonal support that CES1 dysregulation is coupled to chromosomal instability and copy‐number context, while recurrent coding‐sequence mutations in CES1 appear uncommon, strengthening a variant‐to‐biomarker interpretation.

One of the most notable observations is the consistent upregulation of CES1 after both clinical chemoradiotherapy and in vitro irradiation. Such induction suggests that CES1 is part of an acute stress‐adaptive program rather than a passive biomarker. Ionizing radiation imposes profound oxidative and metabolic stress, generating lipid peroxidation products and reactive metabolites that require enzymatic clearance [37–39]. As a carboxylesterase with broad substrate specificity, CES1 is known to hydrolyze lipid esters and detoxify electrophilic intermediates [15, 16, 40]. Its irradiation‐induced upregulation may therefore represent an intrinsic attempt by tumor cells to buffer metabolic damage and maintain homeostasis [41]. Notably, our TCGA‐READ analyses indicate that CES1 expression tracks with copy‐number status, consistent with a gene‐dosage component that may prime baseline CES1 levels and shape the magnitude of stress‐inducible responses. Our findings that CES1 deficiency reduces apoptosis are consistent with this model: when metabolic detoxification is impaired, cells may exhibit a shift in apoptotic threshold through altered redox equilibrium or compensatory survival pathways.

The radioresistant phenotype observed after CES1 knockdown expands current understanding of how metabolic enzymes modulate radiation sensitivity. Traditionally, radioresistance has been associated with enhanced DNA repair, reduced apoptosis priming, or microenvironmental shielding. However, growing evidence suggests that metabolic rewiring profoundly influences radiation response by shaping ROS handling, membrane remodeling, and cell fate decisions [42–44]. CES1 sits directly within this metabolic control layer, suggesting that radiosensitivity may be governed not solely by canonical DNA damage pathways but also by metabolic enzymes that regulate cellular stress thresholds. This introduces a conceptual framework wherein metabolic detoxification capacity functions as a “gatekeeper” determining the transition between survival and apoptosis after irradiation. Notably, DDR and oxidative stress pathways were not directly quantified in the current experiments; thus, these mechanistic links should be interpreted as hypothesis‐generating and warrant targeted validation in future work.

The increased migratory capacity observed in CES1‐silenced cells further supports a broader role for CES1 in restraining aggressive phenotypes. Lipid metabolites are key regulators of membrane fluidity, cytoskeletal dynamics, and EMT‐associated signaling, and dysregulated lipid metabolism has been shown to promote invasion and metastasis across multiple tumor types [45–47]. In this context, loss of CES1 may favor the accumulation of pro‐migratory lipid species or engage downstream lipid‐sensitive transcriptional programs that couple lipid utilization to stemness, EMT, and motility [48]. Importantly, the coexistence of radioresistance and enhanced migration in CES1‐deficient cells is consistent with the broader concept that therapy‐induced stress drives tumor cell plasticity and selects for more aggressive, EMT‐like or stem‐like clones, thereby linking treatment escape with metastatic competence [49, 50]. CES1 deficiency may therefore represent not only a metabolic mechanism of radiation escape but also a node within a plasticity program that shifts tumor cells toward a more malignant, migratory state.

Rescue experiments demonstrate that CES1 reinstatement is sufficient to reverse radioresistance, confirming CES1 as a functional—rather than merely correlative—determinant of radiation response. This has direct translational implications. CES1 is a catalytically active carboxylesterase with well‐characterized roles in xenobiotic metabolism and drug activation, and has already been explored as a pharmacological target in metabolic and oncologic settings [17, 18]. These properties raise the possibility that modulating CES1 activity or stabilizing CES1‐dependent metabolic circuits could be leveraged to enhance radiotherapy efficacy. Importantly, because CES1 coding mutations were infrequent in TCGA‐READ, dysregulation is more plausibly driven by copy‐number and transcriptional mechanisms, suggesting that biomarker translation and therapeutic exploration may prioritize expression/CNV‐informed stratification and pathway‐level modulation rather than mutation‐directed targeting. More broadly, metabolic enzymes are considered more tractable drug targets than transcription factors or structural scaffolds, because their active sites and cofactor‐binding pockets are amenable to small‐molecule intervention [51]. Within this paradigm, a CES1‐restoring strategy—whether through direct enzyme activators, rational metabolic rewiring, or modulation of upstream transcriptional control—may represent an unexplored radiosensitization avenue, conceptually aligned with emerging efforts to target metabolism to overcome radioresistance [43].

Our model also demonstrates the utility of integrating AI‐driven biomarker discovery with mechanistic validation. Machine‐learning algorithms prioritize features based on multivariate interactions that cannot be captured by univariate analysis. CES1 consistently ranked at the top across multiple algorithmic frameworks, underscoring the robustness of its association with radiotherapy response. By linking this computational signature to functional causality, our study bridges the gap between data‐driven discovery and biological mechanism, emphasizing that AI‐derived biomarkers can identify unexpected biologically actionable hypotheses that classical hypothesis‐driven research may overlook. From a clinical perspective, the model may be most useful as a pre‐treatment stratification tool within contemporary nCRT/TNT workflows. Patients predicted to respond favorably could be considered for standard nCRT with response‐adapted management, whereas those predicted to be resistant may be prioritized for TNT intensification, alternative systemic strategies, or clinical trial enrollment. These scenarios remain hypothesis‐generating, and prospective validation will be required to determine whether model‐guided decision support improves outcomes beyond established clinicopathologic and imaging‐based pathways.

Despite these insights, several questions remain. First, the precise metabolic substrates and downstream signaling pathways through which CES1 influences apoptosis and migration require further exploration. Lipidomics and metabolomics profiling will be essential to identify CES1‐dependent metabolic rewiring under irradiation. Second, functional validation was performed in HT‐29 and SW480 cells, which represent distinct molecular backgrounds; thus, extrapolation across broader colorectal cancer subtypes should be made cautiously until additional models are examined. Third, whether CES1 interacts with canonical DNA damage response pathways or modulates radiation‐induced chromatin remodeling remains unknown. Fourth, in vivo studies are needed to determine whether CES1 modulation alters tumor regression, metastatic dissemination, or immune microenvironmental interactions during radiotherapy. In addition, given the low frequency of CES1 coding mutations, pathway enrichment comparisons by CES1 mutation status should be interpreted as descriptive and hypothesis‐generating rather than definitive mechanistic evidence.

In summary, this study uncovers CES1 as a previously unappreciated regulator of rectal cancer radiosensitivity, operating at the confluence of metabolic adaptation, apoptotic priming, and therapy‐induced plasticity. The combined computational and experimental approach highlights CES1 as both a predictive biomarker and a potentially tractable metabolic node with the potential to enhance therapeutic response. By incorporating copy‐number and somatic variation context, our work further supports a mechanistically interpretable route for CES1‐centered biomarker translation in rectal cancer. These findings open new avenues for metabolic radiosensitization strategies and underscore the value of integrating AI‐based discovery with mechanistic oncology research.

## 5. Conclusion

This study identifies CES1 as a regulator of rectal cancer radiosensitivity through integrated multi‐cohort machine learning and functional validation. Genomic profiling in TCGA‐READ indicates that CES1 dysregulation is more consistently associated with copy‐number alteration and transcriptional modulation than with recurrent coding mutations. CES1 appears to influence irradiation response by modulating apoptotic vulnerability and phenotypic plasticity. These findings support CES1 as a predictive biomarker with translational relevance, although further mechanistic and in vivo studies are required before clinical application.

## Author Contributions

XY conceived and designed the study. XY wrote the manuscript. MZ and XL analyzed the data and conducted in vitro experiments. YL helped with the final revision of the manuscript. XY, MZ, and XL contributed equally.

## Funding

No funding was received for this manuscript.

## Disclosure

All authors reviewed and approved the final manuscript.

## Conflicts of Interest

The authors declare no conflicts of interest.

## Data Availability

The data that support the findings of this study are openly available in NCBI Gene Expression Omnibus (GEO) at https://www.ncbi.nlm.nih.gov/geo, reference number GSE46862, GSE35452, GSE94104.
